# Genetic Consequences of Acute/Chronic Gamma and Carbon Ion Irradiation of *Arabidopsis thaliana*

**DOI:** 10.3389/fpls.2020.00336

**Published:** 2020-03-25

**Authors:** Yoshihiro Hase, Katsuya Satoh, Hajime Seito, Yutaka Oono

**Affiliations:** Takasaki Advanced Radiation Research Institute, National Institutes for Quantum and Radiological Science and Technology (QST), Takasaki, Japan

**Keywords:** mutation, gamma ray, ion beam, low dose rate, Arabidopsis

## Abstract

Gamma rays are the most frequently used ionizing radiation in plant mutagenesis; however, few studies are available on the characteristics of mutations at a genome-wide level. Here, we quantitatively and qualitatively characterized the mutations induced by acute/chronic gamma ray irradiation in Arabidopsis. The data were then compared with those previously obtained for carbon ion irradiation. In the acute irradiation of dry seeds at the same effective survival dose, gamma rays and carbon ions differed substantially, with the former inducing a significantly greater number of total mutation events, while the number of gene-affecting mutation events did not differ between the treatments. This may result from the gamma rays predominantly inducing single-base substitutions, while carbon ions frequently induced deletions ≥2 bp. Mutation accumulation lines prepared by chronic gamma irradiation with 100–500 mGy/h in five successive generations showed higher mutation frequencies per dose compared with acute irradiation of dry seeds. Chronic gamma ray irradiation may induce larger genetic changes compared with acute gamma ray irradiation. In addition, the transition/transversion ratio decreased as the dose rate increased, suggesting that plants responded to very low dose rates of gamma rays (∼1 mGy/h), even though the overall mutation frequency did not increase. These data will aid our understanding of the effects of radiation types and be useful in selecting suitable radiation treatments for mutagenesis.

## Introduction

Since the first demonstration of inducing artificial mutations in plants (Stadler, 1928), these mutations have generated various plant genetic resources. The greatest advantage of mutation breeding lies in its ability to improve one or a few specific traits of the preferred variety. In addition, mutation breeding is a simple and ubiquitously applicable technique, and its continued application worldwide indicates its usefulness in plant breeding and biotechnology. In recent years, genome sequences of many plant species have become readily available, and the mutants of genes of interest might be more easily obtained in the future through the use of new breeding techniques, such as genome editing (Schaart et al., 2015). However, it is still difficult to fully identify gene or protein and to select their target genes for breeding purposes based only on their nucleotide sequences. The isolation and analysis of mutants are the most direct and powerful tools to identify gene functions. Thus, the isolation of novel mutants remains an important process.

The gamma ray is the most widely used ionizing radiation in mutagenesis. In fact, 63% of mutant varieties developed using a physical mutagen registered at the Mutant Variety Database (Joint FAO/IAEA) resulted from exposure to gamma rays. Ion beams are energetic particle beams and their application in mutation breeding began in the 1990s in Japan (Tanaka et al., 2010). Their high mutation frequency (MF), wide mutation spectrum and the frequent induction of large deletions and chromosomal rearrangements were reported prior to the middle 2000s (Okamura et al., 2003; Shikazono et al., 2005). Thereafter, ion beams were recognized as useful mutagenic tools. In recent years, ion beam research has been conducted in China and Korea (Du et al., 2017; Kim et al., 2018). Although the use of ion beams is still minor compared with the use of gamma rays, dozens of plant varieties, especially in vegetatively propagated ornamental plants, have been developed to date.

Ion beams are categorized as high-linear energy transfer (LET; the amount of energy that a particle or photon deposits to the material traversed per unit length) radiations, and the LET value varies with the species and energy of the charged particle. Gamma rays are categorized as low-LET radiation. The characterization of the molecular nature of mutations is important for the efficient use of different types of mutagen treatments. Recent advances in high-throughput sequencing technologies have greatly accelerated the understanding of the molecular nature of mutations, particularly those induced by high-LET radiation (Belfield et al., 2012; Hirano et al., 2015; Du et al., 2017; Kazama et al., 2017; Hase et al., 2018; Ichida et al., 2019; Jo and Kim, 2019). An extensive analysis in Arabidopsis suggested that ion beams with very high LET values induce drastic and complex alterations of chromosomes, while ion beams with moderate LET values often induce short insertions and deletions (InDels). Thus, the use of the latter is preferable to achieve a high frequency of loss-of-function mutations (Kazama et al., 2017). We previously compared the mutations induced by carbon ion irradiation of Arabidopsis dry seeds and seedlings, and we demonstrated that the tissue type greatly affected the frequency and types of mutations (Hase et al., 2018). A neutron is also a kind of high-LET radiation, and a large-scale mutant population having a mutation database determined by whole-genome resequencing was constructed in the model rice cultivar Kitaake (Li et al., 2016, 2017). However, limited studies have characterized the mutations induced by gamma rays at a genome-wide level. Shirasawa et al. (2016) performed whole-genome resequencing for three lines of the gamma-irradiated miniature tomato cultivar Micro-Tom and reported that single-base substitutions (SBSs) were the predominant mutations, with the number of gene mutations having a hi- or moderate- impact being 6.3 per line, on average. Recently, Li et al. (2019) compared gamma- and carbon ion-induced mutation in rice using seven mutant lines each and reported that the gamma rays tended to induce larger number of small mutations than carbon ions, whereas the complex structural variations (SVs) were characteristic to the carbon ions. Further characterizations of gamma ray-induced mutations are necessary to reveal the full picture of the mutations induced by different types of ionizing radiation.

Gamma irradiation of plant materials is generally performed in minutes or a few hours with relatively high dose rates (acute irradiation), while irradiation with relatively low dose rates for weeks or months (chronic irradiation) is also used. The facilities known to perform mutation breeding using chronic gamma irradiation are the Gamma Field at the National Agriculture Food Research Organization of Japan (Kawara, 1963, service terminated in June 2019), the Gamma Phytotron at the Korea Atomic Energy Research Institute (Kang et al., 2010) and the Gamma Greenhouse at the Malaysian Nuclear Agency (Azhar and Ahsanulkhaliqin, 2014). Chronic gamma irradiation is believed to induce a wide mutation spectrum with minimum radiation damage (Faiz et al., 2018). Chronic gamma irradiation induced a wider mutation spectrum in carnation than acute gamma irradiation (Okamura et al., 2006). However, little is known about the mutagenic effects of chronic gamma irradiation at the molecular level.

In this study, a whole-genome resequencing analysis was performed to investigate the mutations induced by the acute gamma irradiation of dry seeds and by the chronic gamma irradiation of seedlings in Arabidopsis. For the chronic gamma irradiation, mutation accumulation lines were prepared by recurrent irradiation over five successive generations to evaluate accurately the mutations induced even at low dose rates. The results were compared with the data for carbon ion irradiation obtained in our previous study (Hase et al., 2018) to elucidate how many and what types of mutations are induced and how many genes are affected in a genome by acute/chronic gamma and carbon ion irradiation.

## Results

### Effects of Gamma Irradiation on Plant Growth and the Preparation of Mutation Accumulation Lines

**FIGURE 1:**
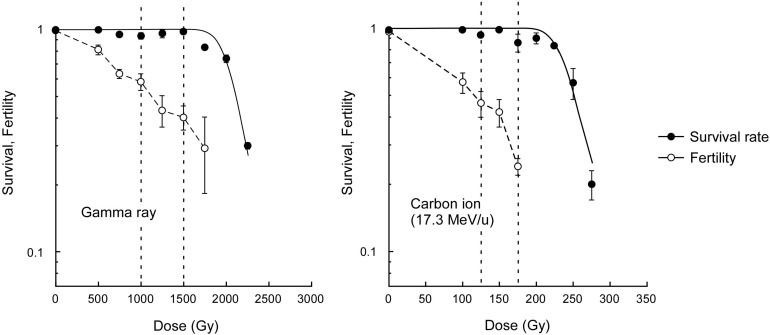
Dose-response relationships for survival rate and fertility of Arabidopsis plants derived from dry seeds irradiated with gamma rays and carbon ions. Closed and open circles represent survival rate and fertility, respectively. Survival curves were drawn on the basis of the single hit-multitarget theory as described in the Materials and methods section. Dotted lines represents 50% and 75% of the dose corresponding to the shoulder of the survival curve (*Dq*). Fertility represents ratio of the number of fertilized ovules to the total number of ovules. Data points are mean ± standard error of three replications with more than 25 plants for survival, and with more than 15 siliques for fertility. Data for carbon ions are from Hase et al. (2018).

The survival rate of dry seeds irradiated with acute gamma rays was examined to determine the dose to compare the mutagenic effects. The dose corresponding to the shoulder of the survival curve (*Dq*) was determined to be 2,045 Gy (Figure 1). Here, based on carbon ion doses used in a previous study (Hase et al., 2018), which corresponded to 75% and 50% of *Dq*, we employed 1,500 and 1,000 Gy, respectively, for the gamma ray irradiation. The M_2_ seeds were collected from individual plants for mutational analysis. Because the *Dq* for carbon ions was 241 Gy, the use of carbon ions reduced the survival by 8.5 times compared with the use of gamma rays per unit dose. The fertility at 75% of *Dq* was significantly lower after carbon ion irradiation than after gamma ray irradiation (*t*-test, *p* < 0.01). This suggests that carbon ions more frequently induce DNA damages that lead to failure during gametogenesis and embryonic lethality than gamma rays.

**FIGURE 2:**
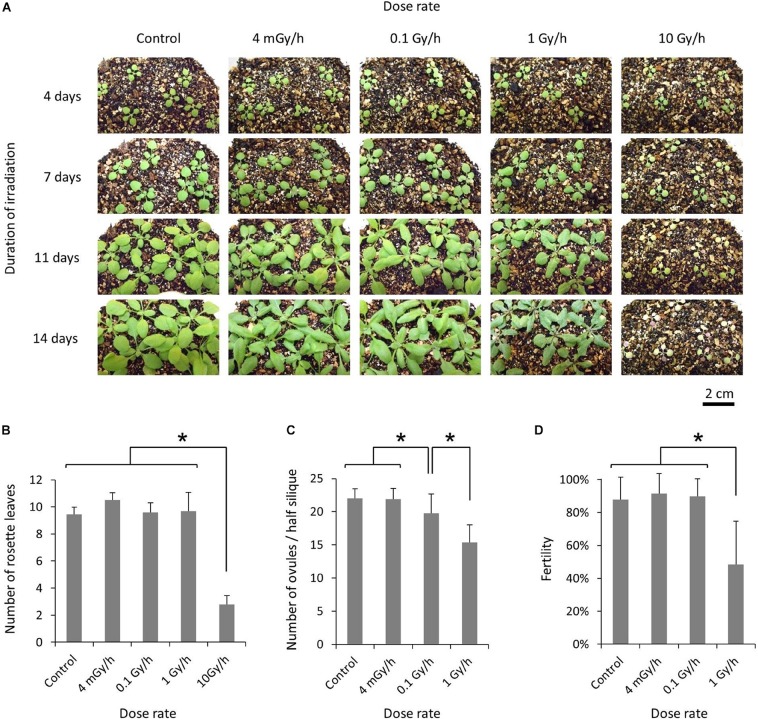
Effects of chronic gamma irradiation on the growth of Arabidopsis plants. **(A)** Plants grown from 7 days after seed sowing under a gamma ray environment with the indicated dose rate and duration. The scale bars represent 2 cm for all the panels. **(B)** Number of rosette leaves per plant examined after the termination of a 14-days irradiation treatment. Data represent means ± standard deviations of 9–10 plants per treatment. **(C,D)** Number of ovules per half silique **(C)** and the percentage of fertilized ovules **(D)**. After a 14-days irradiation treatment, plants were placed in a growth room without gamma irradiation. The total number of ovules and the number of fertilized ovules on one side of the septum were determined for each silique, using the 3rd–5th siliques from the base. Data represent means ± standard deviations of 24–30 siliques collected from 8 to 10 plants per treatment. *Significantly different (*t*-test, *p* < 0.01).

**FIGURE 3:**
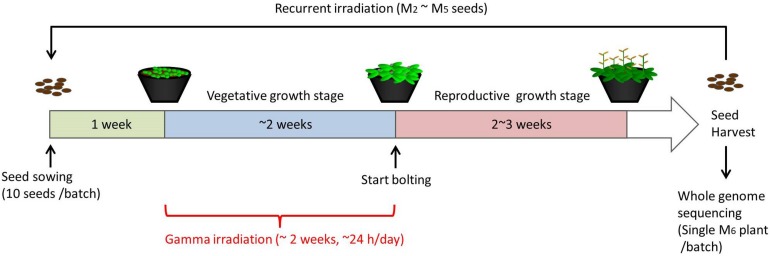
Schematic procedure of generating mutation accumulation lines. Arabidopsis seeds (10 seeds per batch) were sown and grown in a gamma ray environment from 1 week after sowing until plants started bolting (approximately 2 weeks). M_2_ seeds were harvested from each individual batch, and 10 of the M_2_ seeds were used for the next irradiation to obtain M_3_ seeds. This recurrent irradiation was performed until M_6_ seeds were obtained. A single M_6_ plants from each batch was grown and subjected to a whole-genome sequencing analysis.

For chronic gamma irradiation, 7-d-old plants were grown under a gamma ray environment for another two weeks to determine the effects on plant growth. All the plants died without developing new leaves at 10 Gy/h (Figures 2A,B). An apparent growth reduction was not observed at 0.1 Gy/h, although the number of ovules per silique was slightly decreased (Figure 2C). An obvious growth reduction was observed and fertility was halved at 1 Gy/h (Figure 2D). The severe growth reduction and frequently observed sterile siliques suggested that 1 Gy/h is too high a dose rate for mutagenesis. Therefore, mutation accumulation lines were prepared at the dose rates of 500 mGy/h and 73–100 mGy/h. To evaluate the effect of growth environment on mutation induction, mutation accumulation lines were also prepared at 0.6–1.9 mGy/h and 2–4 μGy/h, which is the lowest dose rate that can be prepared in the Gamma Cell. To prepare mutation accumulation lines, one-week-old plants were placed in the Gamma Cell at the four different dose rates and grown for approximately two weeks until the plants started bolting (Figure 3). This irradiation process was repeated in five successive generations, and the M_6_ seeds were used for the whole-genome sequencing analysis. Total doses received during five generations at the dose rates of 500 mGy/h, 73–100 mGy/h, 0.6–1.9 mGy/h and 2–4 μGy/h were 916 Gy, 154 Gy, 2.5 Gy, and 6.2 mGy, respectively. Details of the doses and dose rates are shown in Supplementary Table S1. Control plants were grown in a growth room without gamma irradiation.

A significant reduction in fertility was only observed at 500 mGy/h during the five generations (Supplementary Figure S1A). It is likely that the extent of the reduction plateaued near 30% relative to the control in the 4th and 5th generations. A similar reduction in fertility was observed even when the plants of the 5th generation were grown without gamma irradiation. This suggests that a certain amount of mutations, leading to embryonic lethality, was accumulated in a heterozygous state in the plant population. In this study, mutation accumulation lines were prepared irrespective of the phenotype; however, abnormal pedicel development and fasciation were frequently observed at 500 mGy/h (Supplementary Figures S1B,C). The frequency of those abnormalities was greatly reduced when plants of the 5th generation were grown without gamma irradiation. This suggests that at least most of the abnormal phenotypes represented non-heritable physiological disorders.

### MF and Mutation Types

Whole-genome resequencing was conducted using the M_2_ plants derived from the gamma irradiation of dry seeds and the M_6_ plants of the mutation accumulation lines. In total, 8 randomly chosen independent plants (6 plants in the case of the 1,000-Gy gamma irradiation of dry seeds) were used. The identified mutations were classified into seven categories as described in the Materials and methods section. Data for carbon ions obtained in our previous study (Hase et al., 2018) were reanalyzed for comparison.

**FIGURE 4:**
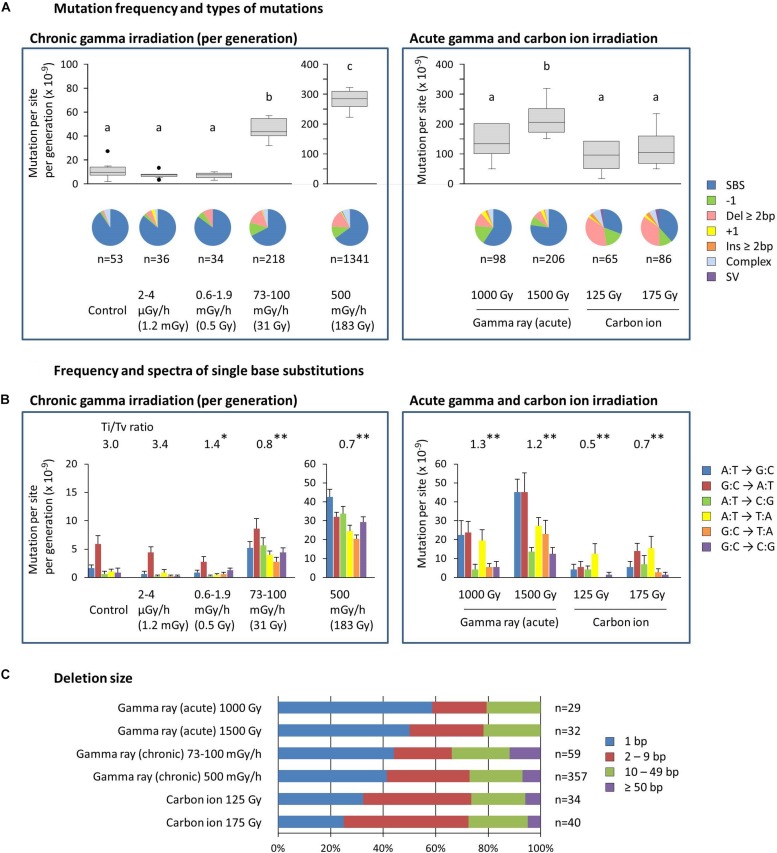
Characterization of the homozygous mutations induced by chronic and acute gamma ray and carbon ion irradiation. **(A)** Mutation frequency per generation and types of mutations. Mutation frequencies are shown with a box and whisker plot. Different letters indicate significant differences in each of the chronic and acute irradiation (one-way ANOVA with multiple comparison test, *p* < 0.05). Numbers below the pie graphs indicate the total number of mutation events identified in each experimental group. Doses in parentheses for chronic gamma irradiation represent the mean total dose received per generation. **(B)** Frequency and spectra of single-base substitutions. Ti/Tv ratios represent the ratios of total transition to transversion events. Asterisks indicate significant differences from the control’s ratio (Chi-squared test, ^∗^*p* < 0.05, ^∗∗^*p* < 0.01). Complementary substitutions (e.g., G:C to A:T and C:G to T:A) were merged. Doses in parentheses for chronic gamma irradiation represent the mean total dose received per generation. **(C)** Ratios of deletion sizes. Numbers indicate the total number of deletions identified in each experimental group.

The mean ratios of homozygous to heterozygous mutations were 0.4–0.7 for dry seed irradiation, and they were not different from the theoretically expected ratio of 0.5 in the M_2_ generation (Supplementary Table S2). The ratios for mutation accumulation lines were 1.1–1.9 because the heterozygous mutation became homozygous as the generations advanced. Here, the characteristics of homozygous mutations were considered because they are thought to be heritable and responsible for most of the mutant phenotypes. The MFs of mutation accumulation lines were divided by five to produce the MF per generation (Figure 4A). Note that this is still overestimated compared with dry seed irradiation because the higher homo/hetero ratio is not being considered. The information on total mutations, including both homozygous and heterozygous mutations, can be found in the supplemental information (Supplementary Figure S2).

The MF of the control, i.e., the spontaneous MF, was 11.1 ± 2.7 × 10^–9^ per site per generation (Figure 4A). SBSs represented more than 90% of the spontaneous mutations, with the G:C to A:T transition being predominant (Figure 4B). These results are comparable with the spontaneous mutations reported in Arabidopsis (MF: 7.1 ± 0.7 × 10^–9^; Ossowski et al., 2010). The MF for gamma irradiation of dry seeds was 1.4–2.2 × 10^–7^, which was 12.4–19.5 times greater than the control (Figure 4A). The MF for carbon ion irradiation of dry seeds was 0.9–1.2 × 10^–7^. Thus, the gamma rays had 1.5–1.8 times greater MFs than the carbon ions at the same effective dose for seed survival. SBSs constituted the major mutation type in gamma ray-treated samples (59%–77% of the total), whereas in carbon ion-treated samples, the ratio of SBSs was about half that of the gamma ray-treated samples (31%–38% of the total) and, instead, a higher ratio of deletions ≥2 bp was observed at 35%–37% compared with 8%–12% in gamma ray-treated samples. The transition/transversion (Ti/Tv) ratio was 3.0 in the control, while it decreased to 1.2–1.3 and 0.5–0.7 in gamma ray- and carbon ion-treated samples, respectively (Figure 4B). This suggests that carbon ions tend to induce a higher ratio of transversions compared with gamma rays.

The MF of chronic gamma irradiation at 73–100 mGy/h was 4.6 × 10^–8^ per site per generation, which was 4.1 times higher than the control (Figure 4A). The highest MF of 2.8 × 10^–7^ was observed at 500 mGy/h. The types of mutations induced by these dose rates were similar to those induced by the acute gamma irradiation of dry seeds. No significant increase in MF was observed at 0.6–1.9 mGy/h and 2–4 μGy/h, and the types of mutations were similar to those of the control. The Ti/Tv ratio tended to decrease as the dose rate increased (Figure 4B). Interestingly, the Ti/Tv ratio at 0.6–1.9 mGy/h was lower than that in the control, despite the non-significant increase in the MF.

Deletion size tended to increase in the following order: acute gamma ray < chronic gamma ray < carbon ion (Figure 4C), and the ratios of deletions ≥2 bp were 41–50%, 56–59%, and 68–75% of the total, respectively. Homozygous deletions larger than 50 bp were observed only in chronic gamma ray- and carbon ion-treated samples. The detailed distribution of deletion size are shown in Supplementary Figure S3.

In our previous study, single-base InDels often occurred in homopolymeric sequences, while InDels ≥2 bp were often associated with polynucleotide repeats or microhomologous sequences (Hase et al., 2018). The mutations induced by acute and chronic gamma irradiation also shared the same features (Supplementary Table S3).

### Impact on Protein-Coding Genes

**FIGURE 5:**
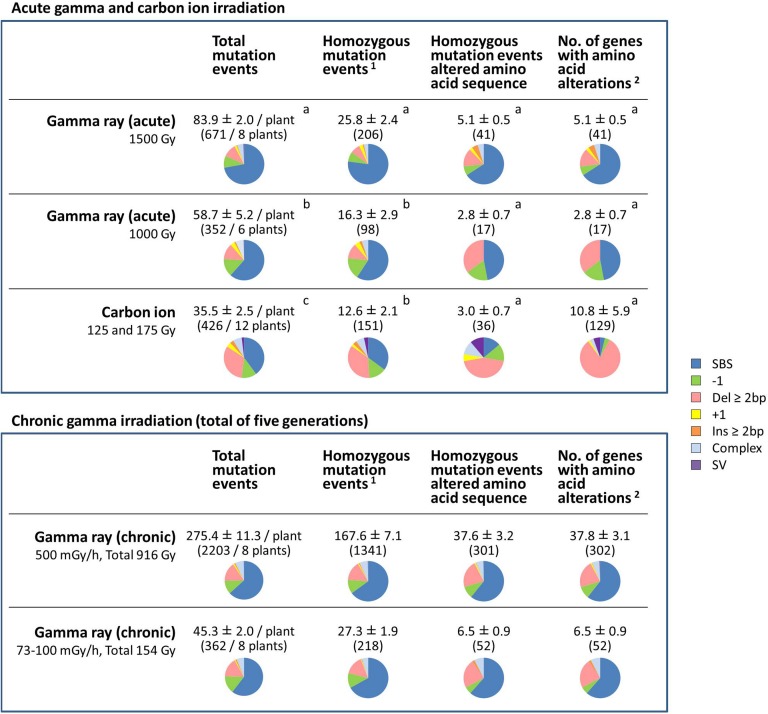
Comparative characterization of the effects of gamma ray- and carbon ion-induced mutation events on protein-coding genes. The values are means ± standard errors of mutation events per plant. The values in parenthesis are total numbers of mutation events. Pie charts represent the types of mutations. Data for carbon ions are from Hase et al. (2018). Different letters indicate significant differences among the acute irradiation treatments in each category (one-way ANOVA with multiple comparison test, *p* < 0.05). ^1^Structural variations including homozygous sequence alterations, which were confirmed by the Integrative Genomics Viewer (IGV), were counted. ^2^Transposable elements, pseudogenes and non-coding RNA were not included.

The effects of different radiation treatments on protein-coding genes were considered (Figure 5). Data for 125 and 175 Gy carbon ion irradiation were merged because there was no significant difference in the MF, and the mutation types were also similar. For the chronic gamma irradiation, total mutation events observed in the M_6_ generation are shown.

For the dry seed irradiation, the numbers of total mutation events, including both homozygous and heterozygous mutations, occurred in the following order: gamma 1,500 Gy > gamma 1,000 Gy > carbon ion 125/175 Gy (Figure 5). The mean number of total mutation events was significantly higher in plants grown from seeds exposed to gamma rays (83.9 per plant at 1,500 Gy; 58.7 per plant at 1,000 Gy) than to carbon ions (35.5 per plant). Of those, the number of homozygous mutation events was significantly higher after gamma 1,500 Gy (25.8 per plant) compared with after gamma 1,000 Gy (16.3 per plant) and carbon ion (12.6 per plant). The number of homozygous mutation events that affected the amino acid sequence ranged from 2.8 to 5.1, and there was no significant difference among the treatments. In case of carbon ion irradiation, more than two genes were occasionally affected by a single mutation event. This was the case in 5 out of 36 homozygous mutation events that affected the amino acid sequence. In particular, 32 and 64 genes were totally lost by 245-kb and 282-kb deletions, respectively. Therefore, there was a large inter-individual difference in the number of affected genes (10.8 ± 5.9 per plant) after receiving carbon ion irradiation. In contrast, no homozygous mutation events affected more than two genes in plants grown from dry seeds exposed to gamma radiation. Homozygous structural variation was observed in 3 out of 12 plants grown from dry seeds that received carbon ions (5 out of 151 homozygous mutation events in total), whereas no homozygous SV was observed in plants grown from dry seeds that received gamma irradiation.

These results suggested three important differences between gamma ray and carbon ion irradiation effects on dry seeds: (1) Gamma rays predominantly induced SBS, whereas carbon ions frequently induced deletion ≥2 bp; (2) Gamma rays induced a significantly higher number of total mutation events, whereas the number of affected genes was not significantly different between plants exposed to the two types of radiation; and (3) Carbon ions induced great inter-individual differences compared with gamma rays.

For chronic gamma ray irradiation, 500 mGy/h (total dose 916 Gy) and 73–100 mGy/h (154 Gy) induced 167.6 and 27.3 homozygous mutation events per plant, respectively. Unlike after the acute gamma irradiation of dry seeds, homozygous SV was observed in 3 out of 8 plants at 500 mGy/h (4 events out of 1,341 homozygous mutation events in total). The predicted structure of SVs are shown in Supplementary Table S4. The mean number of affected genes at 500 mGy/h (37.8 genes per plant) was much higher than after the other treatments. This suggests that recurrent chronic irradiation is effective in accumulating mutations.

## Discussion

In mutant screening, the numbers and types of mutations in a genome are of significance in estimating the MF and the numbers of associated mutations. Here, to provide a more complete picture of the mutations induced by low- and high-LET radiation, we examined the genetic consequences of acute/chronic gamma irradiation and compared the results with data on carbon ion irradiation obtained previously in Arabidopsis (Hase et al., 2018).

For dry seed irradiation, gamma rays induced a higher number of mutation events than carbon ions at the same effective dose based on seed survival (Figures 4, 5). This is consistent with the recent report in rice (Li et al., 2019). The numbers of total mutation events, including both homozygous and heterozygous mutations, were significantly higher at 1.7–2.4-fold, in plants grown from seeds exposed to gamma rays compared with carbon ions (Figure 5). The number of homozygous mutation events was also significantly higher in plants receiving gamma 1,500 Gy than carbon ions. However, the number of homozygous mutation events that affected the amino acid sequence was not significantly different between those receiving gamma rays and carbon ions. Thus, the mutations induced by carbon ions are likely to be more effective in affecting the gene functions than those induced by gamma rays. This could result from the higher ratio of deletions ≥2 bp observed in carbon ion irradiation. For practical mutation breeding, genetic alterations resulting from a small number of mutations is preferable to minimize the number of unnecessary accompanying mutations. Thus, carbon ions may have an advantage over gamma rays in the mutant screening of the M_2_ generation.

The M_2_ populations produced from dry seed irradiation with gamma rays and carbon ions were markedly different in terms of homogeneity. No homozygous mutation events affected more than two genes in plants exposed to gamma rays, whereas in plants exposed to carbon ions, 5 out of 36 homozygous mutation events affected more than two genes, with dozens of genes potentially lost by large deletions. As a result, plants receiving carbon ion irradiation showed large inter-individual differences in the number of affected genes (Figure 5). The involvement of SV was also characteristic of the carbon ion treatments, although the SV ratio of the total homozygous mutation events was not very high (5/151 = 3.3%). Li et al. (2019) also recognized from the comparison between gamma rays and carbon ions in rice that the SVs were characteristic to the carbon ions. Thus, carbon ions appear to induce more drastic genetic changes, with a large inter-individual variability, compared with gamma rays. However, the involvement of large deletions that include more than two genes can make it difficult to identify the genes responsible for a given phenotype. In addition, gamma ray irradiation is advantageous because of the fewer restrictions in sample preparation, a lower cost and more available facilities, compared with ion beam irradiation. The data presented here help in increasing our understanding and selection of suitable radiation treatments for mutant screening.

The chronic gamma irradiation of seedlings showed a higher MF per unit dose compared with acute gamma irradiation of dry seeds. Under our experimental conditions, the total numbers of homozygous mutation events induced by chronic irradiation were approximately 10-fold higher per unit dose than the numbers induced by acute irradiation, at 25.8 mutation events per plant by 1,500 Gy (acute), 16.3 by 1,000 Gy (acute), 27.3 by 154 Gy (chronic), and 167.6 by 916 Gy (chronic) (Figure 5). As mentioned above, because the mutational analysis for chronic irradiation was performed in the M_6_ generation, the number of mutation events was affected by the effects of generation advancement. If we assume that the same number of heterozygous mutations is newly induced in each of the five generations and they are transmitted according to Mendelian inheritance, then the number of homozygous mutation events observed in the M_6_ generation is 8.1 times the number of homozygous mutation events observed in the M_2_ generation (Supplementary Figure S4). Therefore, if the effect of generation advancement was excluded, then the number of mutation events in the M_6_ generation would be 38% lower than the actually observed number. Nevertheless, the chronic gamma irradiation of seedlings resulted in a several times higher MF per unit dose than the acute gamma irradiation of dry seeds. Because the Arabidopsis seedlings were six times more sensitive to carbon ions than dry seeds, as judged by the survival reduction per dose (Hase et al., 2018), the observed difference in MF between chronic and acute gamma irradiation can be explained, in part, by the difference in radiation sensitivity. In addition, chronic gamma irradiation resulted in a smaller fertility reduction than acute gamma irradiation in plants grown under our experimental conditions (Figure 1 and Supplementary Figure S1A). Thus, chronic gamma irradiation appears to be effective in accumulating mutations with minimum radiation damage.

The homozygous SV, which was not detected after acute gamma irradiation, was observed at 500 mGy/h (4 out of 1341 homozygous mutation events = 0.3%), although we cannot conclude that this is specific to chronic gamma irradiation because of the small number of mutation events detected after acute gamma irradiation. This suggests that the frequency of homozygous SV after chronic gamma irradiation is one digit lower than the frequency after carbon ion irradiation of dry seeds (3.3%), although a part of SVs may be overlooked because short-read sequencing was used in this study.

The dry seeds and seedlings are quite different material in term of moisture content and metabolic activities. Generally, high moisture content results in increased irradiation effect via radiolysis of water molecules. Chromatin structure and repair process may also be different in dry seeds and seedlings. In this study, the chronic and acute gamma irradiation were conducted under different experimental conditions including dose, dose rate and material, and therefore, it is difficult to discuss the mechanism that caused the qualitative difference in the induced mutation. Nevertheless, homozygous deletions larger than 50 bp were observed after chronic irradiation, but were not observed after acute gamma irradiation (Figure 4C and Supplementary Figure S3). Thus, chronic gamma irradiation of seedlings may induce relatively larger changes in a genome than acute gamma irradiation of dry seeds. This notion does not contradict with the wider mutation spectrum observed after the chronic gamma irradiation of carnation (Okamura et al., 2006). We previously compared mutation induced by carbon ion irradiation (acute irradiation) on dry seed and seedlings, and showed that dry seed irradiation induced higher ratio of InDels than seedling irradiation (Hase et al., 2018). In contrast, in this study, the types of mutations were similar between acute gamma irradiation of dry seeds and chronic gamma irradiation of seedlings, except the two very low dose rate treatments, in which the MF did not increase (Figure 4A). Since we have not examined the acute gamma irradiation of seedlings, it remains unclear whether those difference is due to radiation quality (carbon ion vs gamma ray) or dose rate (acute vs chronic irradiation), or both. In our previous study, the Ti/Tv ratio was similar between dry seed and seedling irradiation with carbon ions (Hase et al., 2018), however, in this study, the Ti/Tv ratio of acute gamma irradiation of dry seeds (1.2∼1.3) is likely higher than the ratio of chronic gamma irradiation of seedlings (0.7∼0.8) (Figure 4B). This suggests the possibility that DNA damage and/or repair process have qualitative difference in those two different irradiation conditions.

A sucrose pretreatment and carbon ion irradiation of petunia seedlings resulted in a more than two-fold increase in flower color mutant frequency than carbon ion irradiation alone (Hase et al., 2010). Kim et al. (2019) recently reported a similar result for chrysanthemum, in which sucrose and methyl jasmonate pretreatments combined with gamma irradiation increased the mutant frequency by 1.5 times. Thus, the physiological status of the plants may affect the MFs of specific traits. The chronic gamma irradiation process takes more time and effort than that of acute irradiation; however, the former combined with a specific treatment may result in the efficient mutagenesis of plants.

Genetic effects of very low dose/dose rate radiation are also of interest to plant researchers. Over four decades ago, the mutagenic effects of X-rays and gamma rays were assessed using the radiosensitive stamen hairs of *Tradescantia*, and the significant increase in the frequency of somatic mutation was observed after exposure to ∼30 mGy or over (Sparrow et al., 1972; Ichikawa et al., 1981). It was also reported that the effects of 10 mGy/day or less were unlikely to result in any detrimental long-term effects on plant communities, even those of radiosensitive pine trees (International Atomic Energy Agency, 1992). After the accident at the Fukushima Dai-ichi Nuclear Power Plant in 2011, morphological abnormalities were observed in radiosensitive tree species (∼30 μGy/h or less) (Watanabe et al., 2015; Yoschenko et al., 2016). The sharp decrease in the frequencies of abnormalities over time suggested that they represented transient physiological disorders. However, little is known regarding the genetic effects of very low dose radiation at the molecular level. In this study, we showed that the MF did not increase in Arabidopsis exposed to gamma radiation of 0.6–1.9 mGy/h or less (2.5 Gy or less in total) during the vegetative growth stages (Figure 4A). Morphological abnormalities, which are likely to be non-heritable defects, were observed at 73–100 mGy/h and 500 mGy/h in a dose rate-dependent manner (Supplementary Figure S1). Furthermore, the Ti/Tv ratio decreased as the dose rate increased (Figure 4B). The G:C to A:T transition, which is mostly caused by the deamination of methylated cytosines, is a major spontaneous mutation, while an increased frequency of transversion is commonly observed after irradiation due to the generation of DNA damage (Belfield et al., 2012; Li et al., 2016; Du et al., 2017; Kazama et al., 2017; Hase et al., 2018). Because the Ti/Tv ratio at 2–4 μGy/h was comparable with that of the control, the growth environment in the Gamma Cell had a negligible effect on mutation induction. Interestingly, the Ti/Tv ratio at 0.6–1.9 mGy/h was significantly lower than that of the control, despite the non-significant increase in the MF. A similar trend was observed for total mutation events, including both homozygous and heterozygous mutations (Supplementary Figure S2). Willing et al. (2016) reported that wild-type Arabidopsis plants grown under supplemental UVB showed a higher radio of G:C to A:T transition (88%) in all the SBSs compared with the control (52%), despite the total MF was unchanged. They also suggested this is from an increase in the frequency of G:C to A:T transitions in genic regions but not in transposons. Although the underlying mechanism remains unclear, the Ti/Tv ratio might be a good indicator of low dose radiation.

In this study, the mutation accumulation lines were prepared by irradiation during vegetative growth stages. Generally, the reproductive growth stages are more radiosensitive than the vegetative growth stages. In a preliminary experiment, we grew Arabidopsis plants for two weeks from the beginning of bolting at a dose rate of 1.9 mGy/h and analyzed the induced mutations in the following M_2_ generation. The fertility decreased by 30% compared with control plants, suggesting the occurrence of chromosomal aberrations. Whole-genome resequencing of independent plants detected three mutation events per plant on average, with 7 homozygous and 17 heterozygous mutations in total for the 8 plants (Supplementary Table S5). The MF was comparable to the spontaneous mutation frequency; however, the Ti/Tv ratio (7/10 = 0.7) was markedly lower than that for spontaneous mutations. This corroborates that gamma rays at a dose rate of ∼1 mGy/h affect the induction of SBS in Arabidopsis even though the MF is unchanged. In this study, we clearly described the differences in the mutations induced by exposure to acute/chronic gamma rays and carbon ions using the same plant materials and analytical method. We believe the presented data are valuable for improving our understanding of the effects of radiation quality and in selecting a suitable radiation treatment for mutagenesis.

## Materials and Methods

### Plant Material and Growth Conditions

Seeds of *Arabidopsis thaliana* (Columbia accession) obtained from a single plant were used to minimize the background mutations that existed in the laboratory strain. Plants were grown in a growth room (Koito Industries, Yokohama, Japan) at 23°C under a 16-h light/8-h dark photoperiod with ∼70 μmol m^2^/s fluorescent light, unless otherwise indicated.

### Acute Gamma Irradiation

Dry seeds were exposed to ^60^Co gamma rays for one hour at different dose rates to irradiate 500 to 2,250 Gy in Gamma Cell #2 of the Food Irradiation Facility, Takasaki Advanced Radiation Research Institute, National Institutes for Quantum and Radiological Science and Technology. Irradiated seeds were sown in plug trays (200 cells/tray, Takii & Co., Ltd., Kyoto, Japan) filled with a 1:1 mixture of culture soil (TM-2, Takii & Co., Ltd.) and vermiculite (medium size, Vern-piece; Hakugen Co., Ltd., Tokyo, Japan). The survival rate was determined one month after sowing. Three replications of more than 25 seeds were used for each dose. Survival curves were drawn on the basis of the single hit-multitarget theory using the following equation as previously described (Hase et al., 2012):

$$S\,u\,r\,v\,i\,v\,a\,l\,r\,a\,t\,e=1-{\left(1-e^{-D/D_{0}}\right)}^{m},$$

where *D* is the dose, *D*_0_ (the dose conferring 37% survival rate) and m (the extrapolated number) are the parameters. The data were fitted by the least-squares method using the KaleidaGraph (Synergy Software, PA, United States). The shoulder dose (*Dq*) of the survival curves was calculated using the equation:

$$D\,q=D_{0}\times \ln m.$$

Seed fertility was determined as the ratio of the number of fertilized ovules to the total number of ovules as previously described (Hase et al., 2018). Three replications of more than 30 siliques were used for each dose. Seeds of the following generation (M_2_ lines) were collected from individual plants of 1,000 Gy- and 1,500 Gy-irradiated populations undergoing self-pollination.

### Chronic Gamma Irradiation

Dry seeds were sown in a plant box (60-mm W × 60-mm D × 100-mm H, AGC Techno Glass, Shizuoka, Japan) containing a 1:1 mixture of culture soil and vermiculite. Chronic gamma irradiation was carried out in Gamma Cell #1 of the Food Irradiation Facility, Takasaki Advanced Radiation Research Institute, National Institutes for Quantum and Radiological Science and Technology. Dose rates were measured using an ionization chamber (C-110, Applied Engineering, Tokyo, Japan) or a scintillation survey meter (TCS-166, Hitachi-Aloca Medical, Tokyo, Japan). Plants were exposed to gamma rays from 7 d after sowing until the plants started bolting (approximately 2 weeks). The room temperature of the Gamma Cell was kept at 20–22°C. Light was supplied by 60-W fluorescent light bulbs to achieve ∼70 μmol m^2^/s with a 16-h light/8-h dark photoperiod. Plant boxes were weighed at least every 3 days and watered to achieve the original weight. The plants were grown in the growth room before and after the gamma irradiation period. Seed fertility was examined as described above.

Mutation accumulation lines were produced by recurrent chronic gamma irradiation in five successive generations at four different dose rates. In total, 10 seeds were sown in each plant box, and 8 plant boxes were used for each dose rate. Plants were exposed to gamma rays from 7 d after sowing until the plants started bolting. M_2_ seeds were bulk collected from individual plant boxes, and 10 seeds were used for the next radiation treatment to obtain M_3_ seeds. This irradiation was repeated until M_6_ seeds (M_6_ lines) were obtained. Details of the dose rate and total dose are shown in Supplementary Table S1. Controls for the mutation accumulation lines were prepared in the same way, using eight pots (50-mm W × 50-mm D × 50-mm H), in the growth room without gamma irradiation.

### Whole-Genome Sequencing

Six and eight M_2_ lines were randomly chosen for 1,000-Gy and 1,500-Gy acute gamma irradiation, respectively. Eight M_6_ lines each for chronic gamma ray and control were used. Plants were grown in plug trays in the growth room. Single plants were randomly chosen from each line regardless of the phenotype, and genomic DNA was extracted using a MagExtractor Plant Genome DNA extraction kit (Toyobo Co., Ltd., Tokyo, Japan). Sequencing libraries were prepared using a KAPA HyperPlus Kit and SeqCap Adapter Kit (Nippon Genetics Co., Ltd., Tokyo, Japan). The lengths, concentrations, and purity levels of DNA fragments were assessed using an Agilent 2100 Bioanalyzer (Agilent Technologies Japan, Ltd., Tokyo, Japan). Then, 150-bp paired-end reads were obtained using an Illumina NextSeq500 system. The raw paired-end reads were cleaned by removing low quality reads and Illumina adaptor sequences using Trimmomatic (version 0.36^1^). The cleaned data were mapped to the Arabidopsis reference genome (TAIR10.27^2^) using BWA (version 0.7.5^3^), SAMtools (version 1.3.1^4^), and Picard-tools (version 1.119^5^). The candidate mutation sites were identified using GATK Haplotype Caller (version 3.4^6^), Pindel (version 0.2.4^7^), and BreakDancer (version 1.4.5^8^) algorithms. The called raw SBS variants by GATK were filtered using VariantFiltration with parameters of QualByDepth < 2.0, FisherStrand > 60.0, RMSMappingQuality > 40.0, MQRankSum < −12.5, ReadPosRankSum < −8.0 and StrandOddsRatio > 4.0. The called raw InDel variants by GATK were filtered using VariantFiltration with parameters of QualByDepth < 2.0, FisherStrand > 200.0, MQRankSum > 40.0, ReadPosRankSum < −20.0 and StrandOddsRatio > 10.0. The configuration file for BreakDancer was created with the coefficients of variation of 5. The variants ≤10 bp called by Pindel were excluded. The average depth of coverage was 44× for acute gamma irradiation and 37× for chronic gamma irradiation, with ≥99% of the target bases covered at a minimum of 10× (Supplementary Table S6). The candidate mutation sites identified by GATK in more than two independent samples were excluded as background mutations. The candidate mutation sites having allele frequencies (AF; proportions of mutant reads at a site) of 25% or less were also excluded. The AFs showed a reasonable distribution, with two peaks, a sharp peak close to 1.0 (homozygous mutations) and a bilaterally symmetrical broad peak at around 0.5 (heterozygous mutations) after both acute and chronic gamma irradiation treatments (Supplementary Figure S5). All the candidate mutations were confirmed by the IGV (Version 2.4.14^9^). The mutation site was considered heterozygous if 25% < AF < 80%, and if for all the other samples AFs were <5%. The candidate mutation site was considered as homozygous if the AF ≥80%, and if for all the other samples the AFs were <5%. For the Pindel and BreakDancer analyses, the candidate mutation sites unique to a single sample were selected and confirmed by the IGV. The sequence of each read was also confirmed as necessary.

The identified mutations were classified into seven categories as previously described (Hase et al., 2018): (1) SBSs; (2) single-base deletion (−1); (3) deletions of two or more bp (Del ≥ 2 bp); (4) single-base insertions (+1); (5) insertions of two or more bp (Ins ≥ 2 bp); (6) complex types; and (7) SVs. The complex type represents more than two consecutive SBSs, or more than two SBSs and/or short insertion and deletions identified with a gap of less than 10 bases. MF was calculated as the number of mutation events divided by the length of the reference genome. The mutation detection method for carbon ion irradiation was validated by PCR and Sanger sequencing in our previous study (Hase et al., 2018). In addition, here we verified a part of the identified mutations induced by acute/chronic gamma irradiation by PCR and Sanger sequencing. The randomly chosen 33 mutations, which belong to all categories of mutation type, were examined (Supplementary Table S7 and Supplementary Figure S6). Two of them could not be determined because the primers failed to amplify a single specific band with the wild type DNA. The length of the homozygous insertion identified in 500 mGy/h-3 was corrected from 9 bp to 5 bp. The remaining 30 mutations were confirmed to be true. All the identified mutations can be found in the supplemental information (Supplementary Data S1–S3).

## Data Availability Statement

The whole-genome sequencing data analyzed in this study were deposited into the DNA Data Bank of Japan Sequence Read Archive (https://ddbj.nig.ac.jp/dra) with the accession numbers DRA006578 and DRA008787.

## Author Contributions

YH designed and performed the experiment, interpreted the data, and wrote the manuscript. HS prepared the gamma irradiation field. KS analyzed the raw sequence data. YO managed the project. All authors commented and approved the final manuscript.

## Conflict of Interest

The authors declare that the research was conducted in the absence of any commercial or financial relationships that could be construed as a potential conflict of interest.

## Footnotes

1 http://www.usadellab.org/cms/?page=trimmomatic

2 https://www.arabidopsis.org/

3 http://bio-bwa.sourceforge.net/

4 http://samtools.sourceforge.net/

5 https://broadinstitute.github.io/picard/

6 https://software.broadinstitute.org/gatk/

7 http://gmt.genome.wustl.edu/packages/pindel/user-manual.html

8 http://breakdancer.sourceforge.net/

9 http://software.broadinstitute.org/software/igv/
