# Supplementary material for: Genetic Consequences of Acute/Chronic Gamma and Carbon Ion Irradiation of Arabidopsis thaliana
Source: Front Plant Sci. 2020 Mar 25;11:336. doi: 10.3389/fpls.2020.00336 (PMC7113374; doi:10.3389/fpls.2020.00336)
Supplement: Supplementary file 4 [file Data_Sheet_1.pdf]

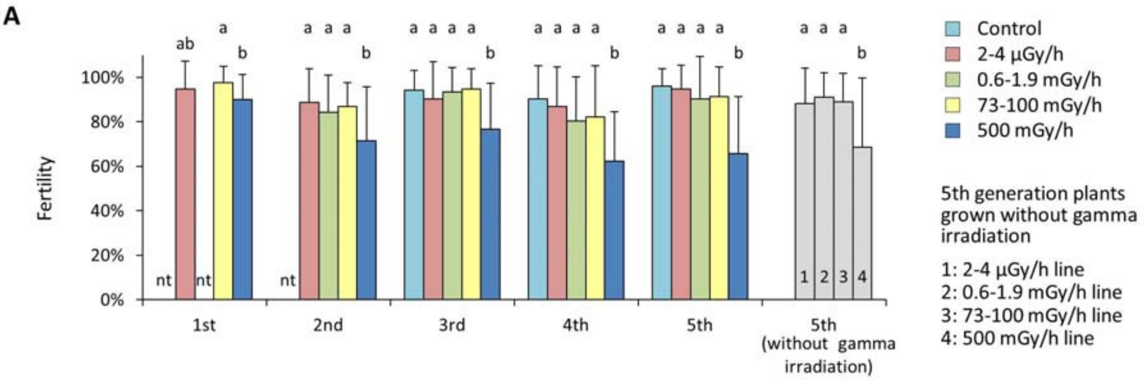

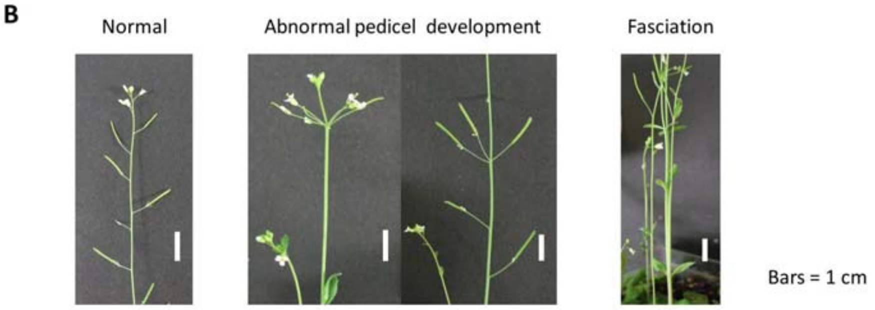

C

Irradiated population of 4th generation

	Control	73-100 mGy/h	500 mGy/h
Abnormal pedicel development	0% (0/40)	5% (3/57)	100% (45/45)
Fasciation	0% (0/40)	0% (0/57)	7% (3/45)

Irradiated population of 5th generation

	Control	73-100 mGy/h	500 mGy/h
Abnormal pedicel development	0% (0/30)	0% (0/57)	93% (41/44)
Fasciation	0% (0/30)	0% (0/57)	11% (5/44)

Plants grown without gamma irradiation

	73-100 mGy/h	500 mGy/h
Abnormal pedicel development	0% (0/30)	7% (2/30)
Fasciation	0% (0/30)	0% (0/30)

Supplementary Figure S1. Effects of chronic gamma ray irradiation on fertility and abnormal developmental phenotypes. (A) Fertility observed at each dose rate and for each generation during preparation of mutation accumulation lines. Data represent means ± standard deviations. Different letters indicate significant differences in each generation (one-way ANOVA with multiple comparison test, $p < 0.05$). nt: not tested. (B) Abnormal developmental phenotypes observed in the irradiated plants. (C) Frequency of abnormal phenotype observed in the 4th and 5th generations with or without gamma ray irradiation.

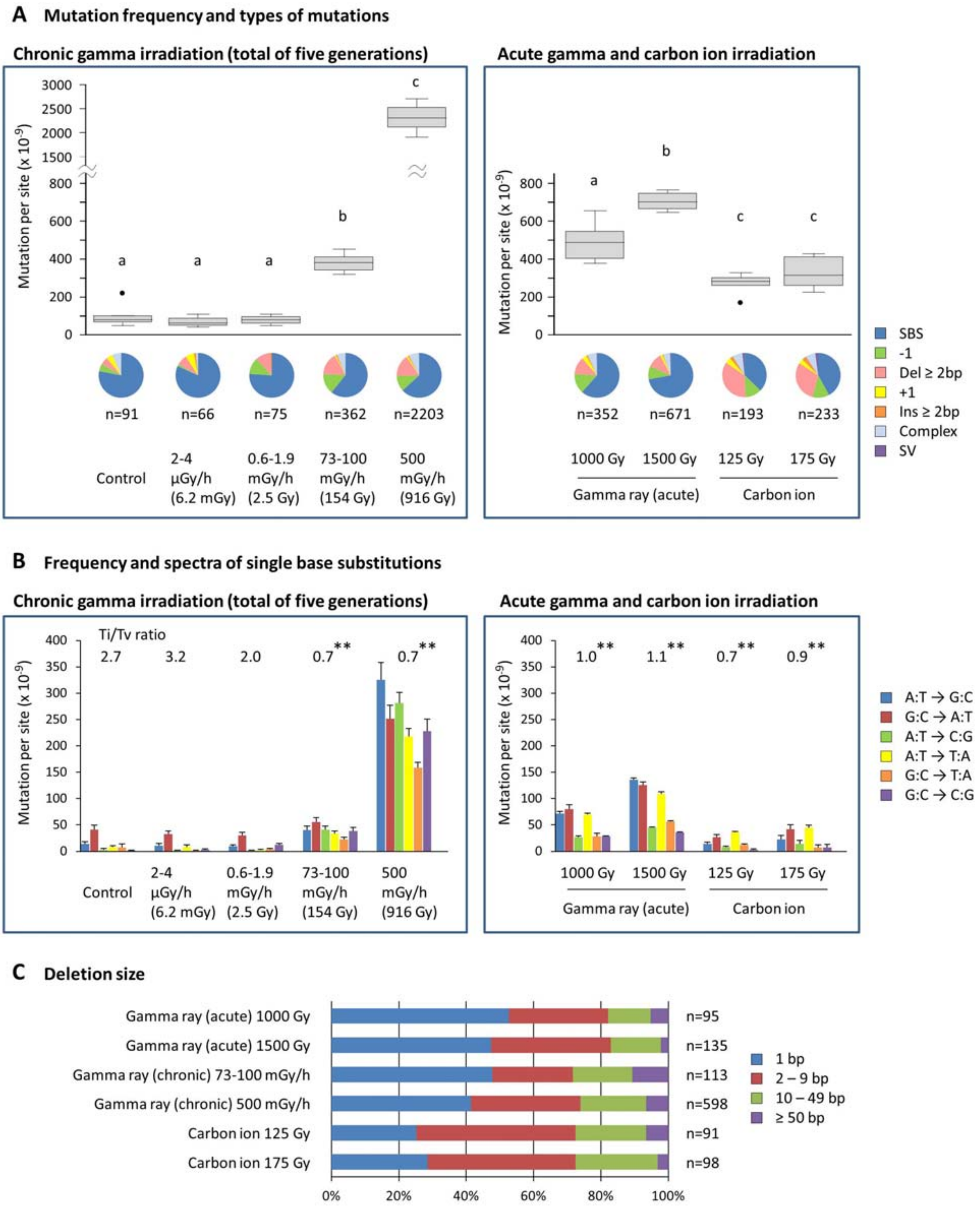

Supplementary Figure S2. Characterization of total mutations, including both homozygous and heterozygous mutations, induced by chronic and acute gamma ray and carbon ion irradiation. (A) Mutation frequency and types of mutations. Mutation frequencies are shown using a box and whisker plot. Different letters indicate significant differences in each of the chronic and acute irradiation (one-way ANOVA with multiple comparison test, $p < 0.05$). Numbers below the pie graphs indicate total numbers of mutation events identified in each experimental group. Doses in parentheses for chronic gamma ray irradiation represent total doses received over five generations. (B) Frequencies and spectra of single-base substitutions. The Ti/Tv ratio represents the ratio of total transitions to transversions. Asterisks indicate significant differences from the ratio of the control (Chi-squared test, ** $p < 0.01$). Complementary substitutions (e.g., G:C to A:T and C:G to T:A) are merged. Doses in parentheses for chronic gamma ray irradiation represent total doses received over five generations. (C) Ratio of deletion size. Numbers indicate the total number of deletions identified in each experimental group.

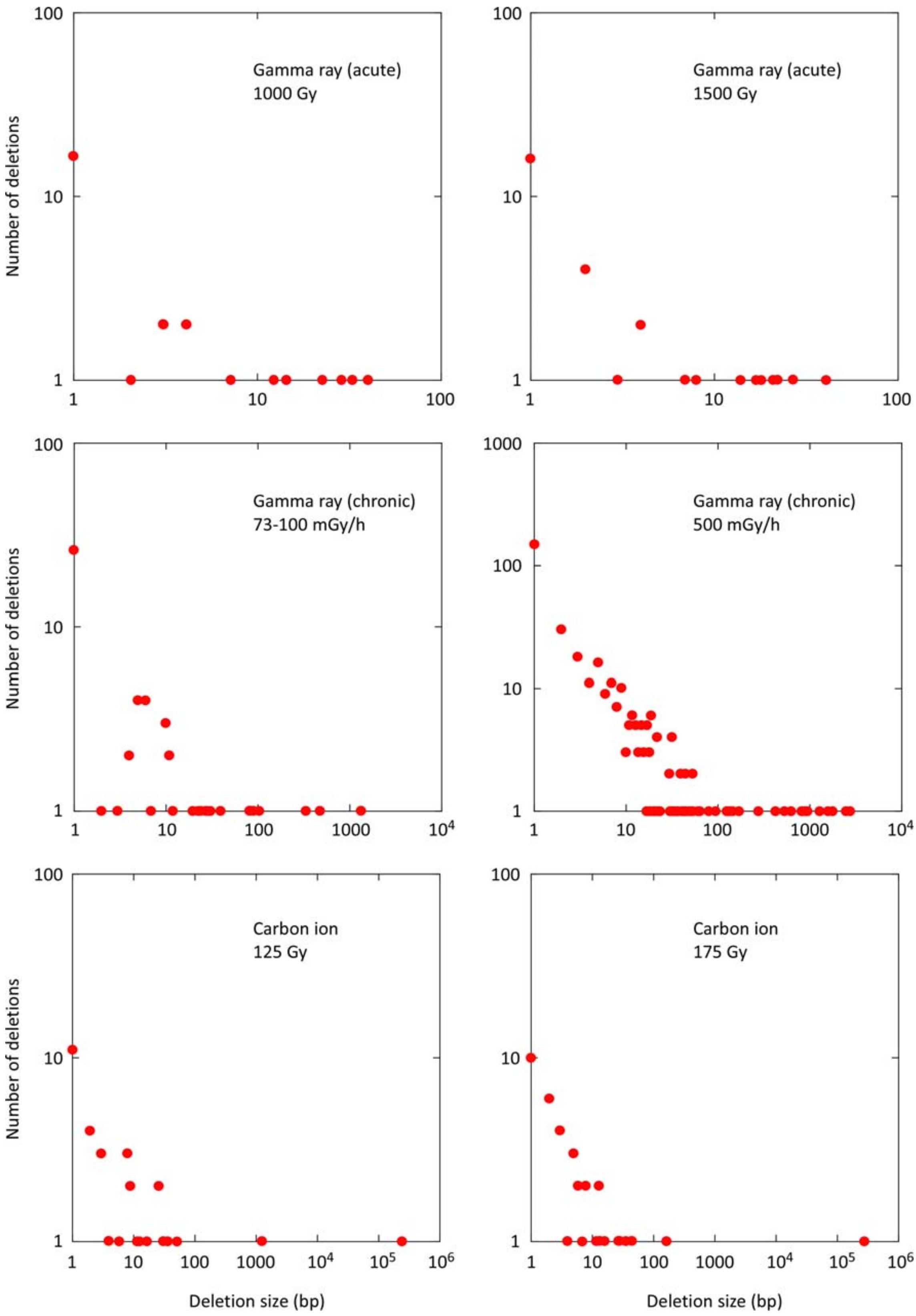

Supplementary Figure S3. Distribution of the size of homozygous deletion.

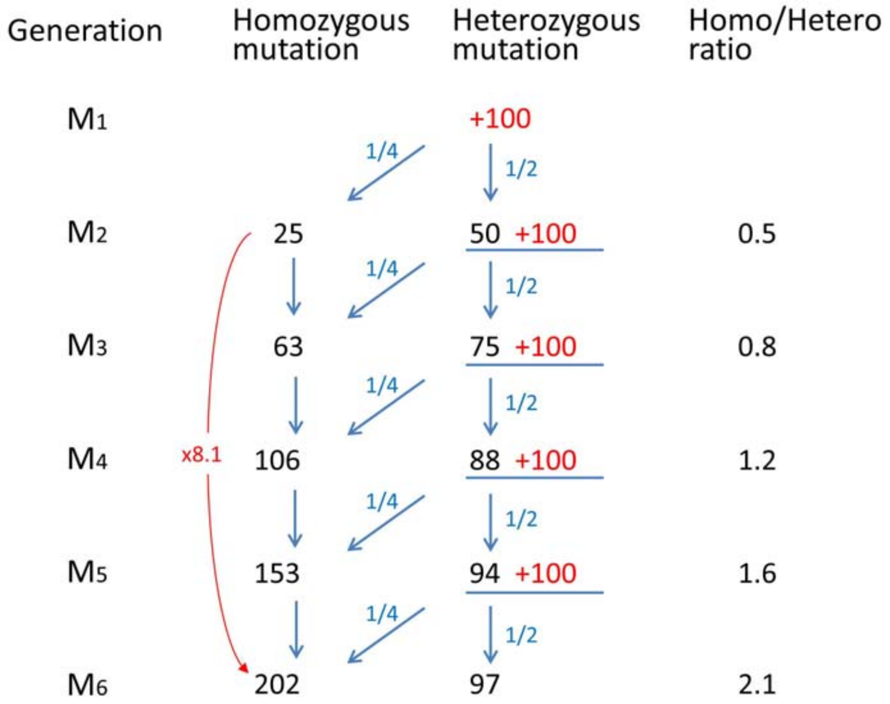

Supplementary Figure S4. Estimation of the effects of generation advancement on the number of homozygous mutations in the mutation accumulation line. Assuming that the same number of heterozygous mutations were newly induced in each generation and transmitted according to Mendelian inheritance, the number of homozygous mutation events observed in the M_6 generation is 8.1 times that of the number of homozygous mutations observed in the M_2 generation. If the effect of generation advancement was excluded, the number of homozygous mutation events in the M_6 generation would be 125 (25×5 generations), which is 38% lower than the actually observed number (202).

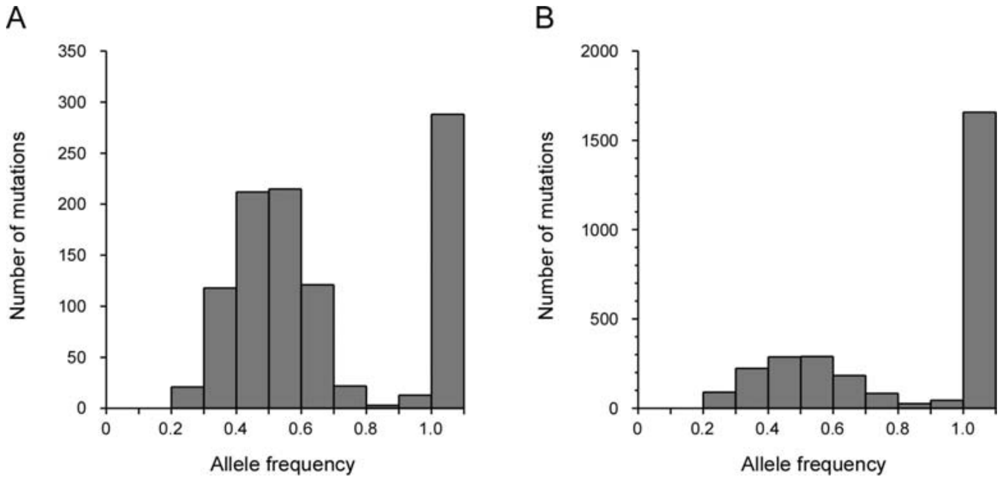

Supplementary Figure S5. Distribution of allele frequencies for mutations detected using the GATK algorithm. In total, (A) 1,013 mutations were derived from acute gamma ray irradiation of dry seeds, and (B) 2,885 mutations were derived from chronic gamma ray irradiation during the vegetative growth stage in five successive generations.

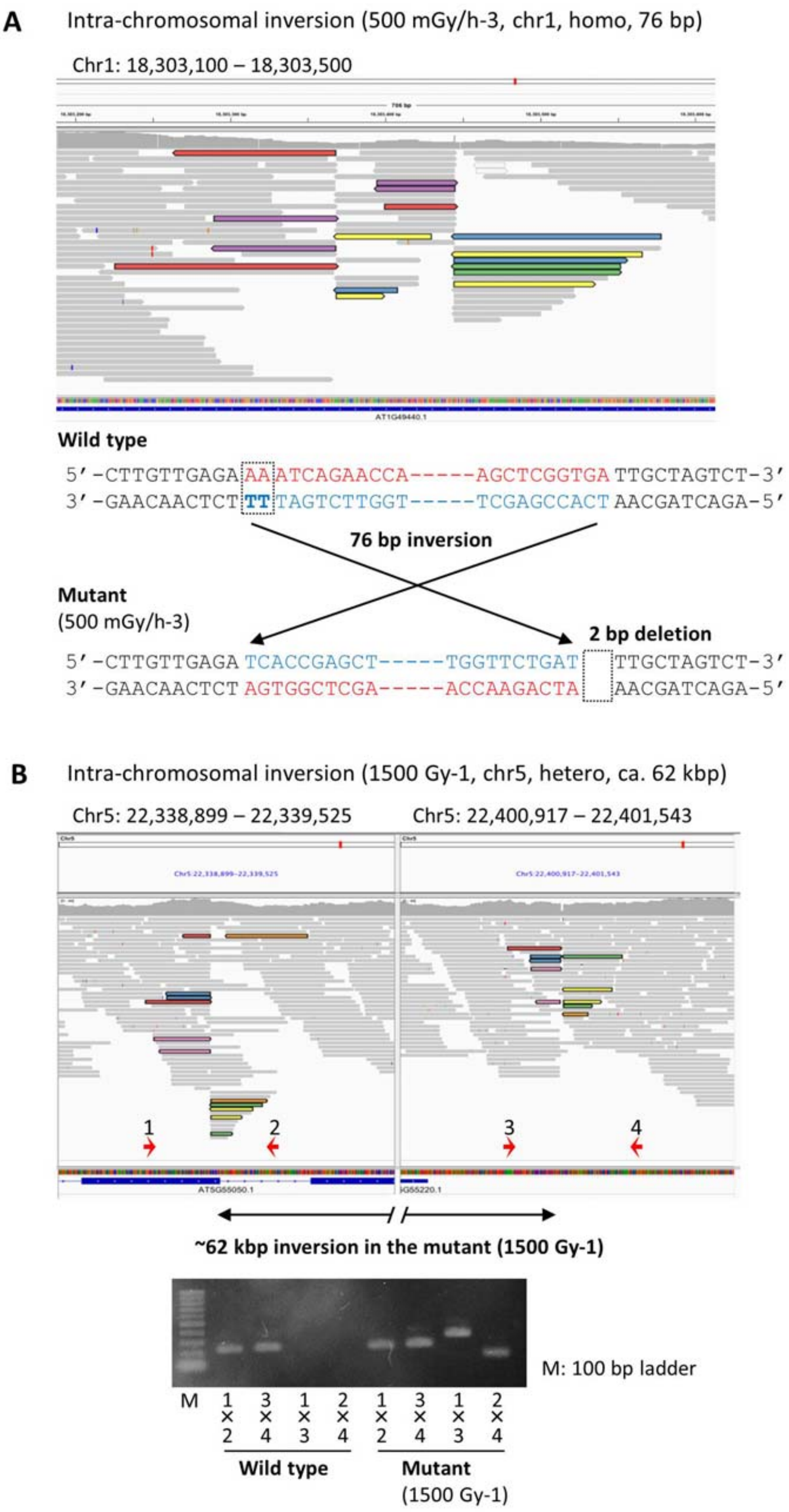

Supplementary Figure S6. Structural variations confirmed by PCR and Sanger sequencing. (A) Intra-chromosomal inversion of 76 bp identified in 500 mGy/h-3. Several pairs of the sequence reads are indicated in different colors. (B) Intra-chromosomal inversion of ca. 62 kbp identified in 1500 Gy-1. Arrows in the snapshot of IGV browser indicates the position and direction of PCR primers. The gel image shows PCR products amplified with the indicated primer pairs. The primer sequences used here are shown in Supplemental Table 7.

Supplementary Table S1. Dose rate and total dose of gamma ray during preparation of mutation accumulation lines.

Generation	Total irradiation time	Dose rate (mGy/h) (Total dose (Gy))			
		Group 1	Group 2	Group 3	Group 4
1st	351h 44m<br>(Jul 21 - Aug 5, 2015)	0.0022<br>(0.00077)	0.57<br>(0.2)	100<br>(35)	500<br>(176)
2nd	351h 8m<br>(Oct 1 - Oct 16, 2015)	0.0022<br>(0.00077)	0.55<br>(0.2)	100<br>(35)	500<br>(176)
3rd	377h 44m<br>(Mar 15 - Mar 31, 2016)	0.0043<br>(0.0016)	1.9<br>(0.7)	77<br>(29)	500<br>(189)
4th	353h 48m<br>(Jun 15 - Jul 1, 2016)	0.0042<br>(0.0015)	1.9<br>(0.7)	74<br>(26)	500<br>(177)
5th	395h 24m<br>(Aug 26 - Sep 12, 2016)	0.0041<br>(0.0016)	1.8<br>(0.7)	73<br>(29)	500<br>(198)
Total	1,830h 48m	(0.0062)	(2.5)	(154)	(916)

Supplementary Table S2. Zygosity of identified mutations.

Acute irradiation on dry seeds.

	Gamma ray (acute)		Carbon ion	
	1000 Gy	1500 Gy	125 Gy	175 Gy
Homo / hetero ratio	0.4 ± 0.2	0.5 ± 0.2	0.6 ± 0.4	0.7 ± 0.6

Numbers are mean ± standard deviations. There was no significant difference from the expected ratio of 0.5 in the M_2 generation (*t*-test).

Chronic gamma irradiation in the vegetative growth stage.

	Control	Gamma ray (chronic)			
		2~4 μGy/h	0.6~1.9 mGy/h	73~100 mGy/h	500 mGy/h
Homo / hetero ratio	1.8 ± 1.1	1.7 ± 1.4	1.1 ± 0.9	1.6 ± 0.4	1.9 ± 1.0

Numbers are mean ± standard deviations. There was no significant difference from the control's ratio (*t*-test).

Supplementary Table S3. Characteristics of sequence at mutation sites or rejoined sites of insertion and deletion mutations. Numbers in parenthesis indicate number of mutation events associated with homopolymeric sequences (≥3 bp, e.g. AAAA), polynucleotide repeats (e.g. TATA) or microhomology at mutation site or rejoined site / total number of mutation events. Data for carbon ions are modified from Hase et al. (2018).

	Gamma ray 1000 Gy		Gamma ray 1500 Gy		Carbon ion 125 Gy, 175 Gy	
Type of Mutation	Homopolymeric sequence	Polynucleotide repeat or microhomology	Homopolymeric sequence	Polynucleotide repeat or microhomology	Homopolymeric sequence	Polynucleotide repeat or microhomology
-1	42% (20/48)	17% (8/48)	41% (25/61)	11% (7/61)	43% (21/49)	16% (8/49)
Del ≥2 bp	10% (4/42)	55% (23/42)	11% (7/63)	43% (27/63)	9% (12/129)	49% (63/129)
+1	42% (5/12)	17% (2/12)	50% (4/8)	13% (1/8)	43% (6/14)	21% (3/14)
Ins ≥2 bp	0% (0/4)	75% (3/4)	25% (1/4)	75% (3/4)	8% (1/13)	69% (9/13)

	Gamma ray 73~100 mGy/h (154 Gy)		Gamma ray 500 mGy/h (916 Gy)	
Type of Mutation	Homopolymeric sequence	Polynucleotide repeat or microhomology	Homopolymeric sequence	Polynucleotide repeat or microhomology
-1	58% (31/53)	11% (6/53)	47% (114/245)	7% (16/245)
Del ≥2 bp	4% (2/57)	40% (23/57)	7% (22/335)	39% (132/335)
+1	67% (2/3)	0% (0/3)	59% (13/22)	5% (1/22)
Ins ≥2 bp	50% (2/4)	50% (2/4)	7% (1/15)	87% (13/15)

Supplementary Table S4. Predicted structural variations caused by acute/chronic gamma irradiation.

Acute gamma irradiation on dry seeds.

Sample	Zygosity	Deduced structural variation
1500 Gy-1	hetero	intra-chromosomal inversion (chr5, ca. 62 kbp) *
1500 Gy-3	hetero	intra-chromosomal inversion (chr3, ca. 105 kbp)
1500 Gy-4	hetero	intra-chromosomal inversion (chr2, ca. 159 kbp)
1000 Gy-3	hetero	intra-chromosomal inversion (chr4, ca. 3,863 kbp)

Chronic gamma irradiation in the vegetative growth stage.

Sample	Zygosity	Deduced structural variation
500 mGy/h-3	homo	intra-chromosomal inversion (chr1, 76 bp) *
500 mGy/h-6	homo	intra-chromosomal inversion (chr5, ca. 571 kbp)
500 mGy/h-6	homo	intra-chromosomal inversion (chr5, ca. 400 bp)
500 mGy/h-7	hetero	intra-chromosomal inversion (chr5, ca. 2,100 bp)
500 mGy/h-7	homo	intra-chromosomal inversion (chr5, ca. 800 bp)

*Details are shown in Supplemental Figure 6.

Supplementary Table S5. Summary of the mutations detected after chronic gamma ray irradiation during reproductive growth stage.

Type of mutation		Homozygous	Heterozygous	Total
Single base substitution				
Transition	A:T → G:C	0	1	1
	G:C → A:T	0	6	6
Transversion	A:T → C:G	0	1	1
	A:T → T:A	0	2	2
	G:C → T:A	4	2	6
	G:C → C:G	0	1	1
Deletion	1 bp	1	0	1
	≥ 2 bp	2	0	2
Insertion	1 bp	0	2	2
	≥ 2 bp	0	1	1
Complex		0	1	1
Structural variation		0	0	0
Total		7	17	24

The wild-type Arabidopsis plants were grown in the Gamma Cell for two weeks from the beginning of bolting at a dose rate of 1.9 mGy/h, and M_2 seeds were harvested from individual plants. Whole genome resequencing and mutational analysis were conducted in the same way for the other samples in this study. Data are from eight independent M_2 plants.

Supplementary Table S6. Summary of mapping results.

Acute gamma irradiation on dry seeds.

Sample	Total mapped base (Mb)	Mean depth of coverage	Coverage above 10x (%)	Coverage above 25x (%)
Gamma 1000 Gy				
1000Gy-1	4,876	40.8	99.5	72.9
1000Gy-2	5,944	49.8	99.8	90.6
1000Gy-3	5,546	46.4	99.7	83.8
1000Gy-4	5,071	42.4	99.6	74.7
1000Gy-5	4,586	38.4	99.4	60.9
1000Gy-6	5,072	42.5	99.6	75.8
Gamma 1500 Gy				
1500Gy-1	5,405	45.2	99.7	82.9
1500Gy-2	5,221	43.7	99.6	77.6
1500Gy-3	5,052	42.3	99.5	72.5
1500Gy-4	5,777	48.4	99.7	86.4
1500Gy-5	5,341	44.7	99.7	79.7
1500Gy-6	4,860	40.7	99.5	66.8
1500Gy-7	5,321	44.5	99.7	80.6
1500Gy-8	5,947	49.8	99.8	90.6
Mean	5,277	44.2	99.6	78.3
SD	384	3.2	0.1	7.9

Chronic gamma irradiation in the vegetative growth stage.

Sample	Total mapped base (Mb)	Mean depth of coverage	Coverage above 10x (%)	Coverage above 25x (%)
Control				
Control-1	4,126	34.5	98.9	50.5
Control-2	4,144	34.7	99.0	51.6
Control-3	4,285	35.9	99.1	54.7
Control-4	3,934	32.9	98.6	45.8
Control-5	3,718	31.1	98.3	44.7
Control-6	5,252	44.0	99.6	85.1
Control-7	5,295	44.3	99.7	85.7
Control-8	4,178	35.0	99.1	61.1

Continued on the next page.

Supplementary Table S6 – continued.

Sample	Total mapped base (Mb)	Mean depth of coverage	Coverage above 10x (%)	Coverage above 25x (%)
Gamma 500 mGy/h				
500mGyh-1	4,346	36.4	99.3	61.3
500mGyh-2	4,179	35.0	98.8	61.8
500mGyh-3	4,509	37.7	98.7	46.4
500mGyh-4	4,686	39.2	99.5	67.8
500mGyh-5	4,052	33.9	99.0	55.2
500mGyh-6	3,078	25.8	94.4	17.9
500mGyh-7	4,459	37.3	99.3	64.4
500mGyh-8	4,825	40.4	99.6	78.2
Gamma 73~100 mGy/h				
100mGyh-1	4,540	38.0	99.4	64.9
100mGyh-2	3,977	33.3	98.5	43.4
100mGyh-3	4,731	39.6	99.2	64.2
100mGyh-4	3,708	31.0	98.3	40.2
100mGyh-5	4,074	34.1	99.0	56.1
100mGyh-6	3,748	31.4	98.0	47.6
100mGyh-7	3,816	31.9	98.4	43.9
100mGyh-8	4,848	40.6	99.5	70.5
Gamma 0.6~1.9 mGy/h				
06mGyh-1	4,628	38.7	99.4	66.5
06mGyh-2	3,769	31.6	96.8	35.8
06mGyh-3	5,634	47.2	99.8	87.1
06mGyh-4	4,054	33.9	98.6	48.2
06mGyh-5	5,011	41.9	99.7	82.4
06mGyh-6	4,680	39.2	99.5	77.6
06mGyh-7	4,786	40.1	99.6	77.2
06mGyh-8	5,783	48.4	99.8	91.4
Gamma 2~4 μGy/h				
2μGy-1	4,580	38.3	99.2	60.4
2μGy-2	4,265	35.7	98.5	44.1
2μGy-3	4,538	38.0	99.1	59.8
2μGy-4	5,228	43.8	99.6	73.8
2μGy-5	4,927	41.2	99.6	78.7
2μGy-6	4,613	38.6	99.5	72.9

Continued on the next page.

Supplementary Table S6 – continued.

Sample	Total mapped base (Mb)	Mean depth of coverage	Coverage above 10x (%)	Coverage above 25x (%)
2μGy-7	4,002	33.5	98.9	49.8
2μGy-8	4,694	39.3	99.6	75.3
Mean	4,443	37.2	99.0	61.1
SD	562	4.7	1.0	16.3

Chronic gamma irradiation in the reproductive growth stage.

Sample	Total mapped base (Mb)	Mean depth of coverage	Coverage above 10x (%)	Coverage above 25x (%)
Gamma 1.9 mGy/h				
1.9mGy-1	5,163	43.2	99.6	77.6
1.9mGy-2	6,349	53.1	99.8	93.7
1.9mGy-3	6,299	52.7	99.8	94.2
1.9mGy-4	5,620	47.0	99.8	87.4
1.9mGy-5	5,305	44.4	99.6	78.5
1.9mGy-6	5,540	46.4	99.7	85.1
1.9mGy-7	5,555	46.5	99.7	86.6
1.9mGy-8	5,825	48.8	99.8	90.3
Mean	5,707	47.8	99.7	86.7
SD	430	3.6	0.1	6.2

Supplementary Table S7. Results of verification of identified mutations induced by acute/chronic gamma irradiation.

Sample	Chr#	Position	Types of mutation	Zygosity	Detail of mutation	Forward and reverse primer (5'→3')	Result of validation
500mGy/h-3	1	666,239	SBS	homo	C to A	F: GGTTTTGTGTTCACCGGTTT<br>R: GGAAACGTGAGGCAGAAGTT	True
500mGy/h-3	1	1,310,801	SBS	hetero	G to C	F: ACGATCAATCGTCGTTACCA<br>R: GTGTCGGTGCCTCAGCTAAT	True
500mGy/h-3	1	1,388,201	SBS	homo	G to T	F: AACGTTCCAAAGGCTTACCA<br>R: TGGCCTGAATGTTTCTGTCA	True
500mGy/h-3	1	5,742,162	SBS	hetero	A to C	F: CAAATGATGCACCAAAACCA<br>R: ACAGCATGGGTCGAGTCTTC	True
500mGy/h-3	1	4,107,178	-1	homo	C	F: TCGACCATACAGCGAAACTG<br>R: TTTCCGACTAATTCCGAGAGA	True
500mGy/h-3	1	4,247,558	-1	hetero	T	F: AAATGCAGAAGCAGCTCAAAA<br>R: ACCCGAACCCGAAATTAAAC	Undetermined (No band amplified in the wild type)
500mGy/h-3	2	2,953,591	+1	homo	A	F: CCACGCTACACGTGGGTATT<br>R: ATCAAACTGGAGTGGGTTGC	Undetermined (Multiple bands amplified in the wild type)
500mGy/h-3	3	3,051,158	+1	hetero	A	F: AGAAAAATGGCCATCCCCTA<br>R: CGAAGCTTGCCAAAGGATAG	True
500mGy/h-3	1	1,416,545	Deletion	homo	-7	F: TCAAGACAAAACCCATGTGC<br>R: GCTTGGCCAACAACAAAGTT	True
500mGy/h-3	1	5,386,271	Deletion	homo	-43	F: CCTCAGCAAATTGGATAACGA<br>R: TTGCCAAATCCATACAATCG	True

Continued to the next page.

Supplementary Table S7. -continued.

500mGy/h-3	1	10,030,954	Deletion	hetero	-4	F: ATCTCCATGGAATGCTCCAG<br>R: CTTTCTGTTCCCGCTCTCAA	True
500mGy/h-3	1	24,024,048	Deletion	hetero	-24	F: CAGGCAACCGTGATAAATGA<br>R: TAGCCGAGATCCACAAGTCA	True
500mGy/h-3	2	7,188,477	Insertion	homo	+3	F: AAGAGTGCTTGTGGCCTTGT<br>R: CCGTTCATCAGATGGCTTTT	True
500mGy/h-3	4	846,911	Insertion	homo	~~+9(-25+34)~~<br>+5(-25+30)	F: GGAGAGCAATTGATGGGGTA<br>R: GCTTTGACTCATACTCTCTTTCGAC	Data corrected*
500mGy/h-3	1	14,317,256	Complex	homo	SBSx2	F: TGAAATGGGTGACGAACAGA<br>R: AACGCTCCCATGTTAGGTTG	True
500mGy/h-3	4	18,187,196	Complex	homo	-1(-4+3),<br>-22(-26+4)	F: CAAGGGGGAAGAACATTGAG<br>R: CTTGCACTTTGAGCAACCAA	True
500mGy/h-3	5	8,601,847	Complex	hetero	-9, +1, -4	F: TCATCGAATCAATAACACATGG<br>R: GACGGGTTTGACAAGACGTT	True
500mGy/h-3	1	18,303,368	SV	homo	76 bp<br>inversion	F: GCTGGATCCCTACTTGTTCG<br>R: GGTCAGAGTCCGACTAAAGAAA	True
Details are shown in Supplemental Figure 6.							
1500Gy-1	1	301,575	SBS	homo	A to T	F: GCATCCAGCCAAAATTTGAT<br>R: TTTCCGATCTTCCGTTTTTG	True
1500Gy-1	1	2,160,037	SBS	homo	C to T	F: AGCATAAAACGCACCAATCC<br>R: GCTCACGCCGTTTACTTCTC	True
1500Gy-1	1	7,421,082	SBS	hetero	A to C	F: ATCGGCCCAATATGGTTAGC<br>R: CTTTCGTCTTCTCCGGTCAG	True
1500Gy-1	1	8,282,971	SBS	hetero	T to C	F: TCGACGTCAATACCACACTTCT<br>R: GCATCAAGGTTGACAATCCA	True

Continued to the next page.

Supplementary Table S7. -continued.

1500Gy-1	1	8,842,662	-1	hetero	T	F: CGCACTCACACTCACACTCA<br>R: GAAAAAGCAAAACAATTAAACCAAA	True
1500Gy-1	1	17,975,131	-1	hetero	G	F: GGGTCTTCCTACGGGTTTTC<br>R: AGAGAGTTGACGCTGGTGGT	True
1500Gy-1	2	7,935,710	-1	homo	T	F: TTGTTCGAAGATGGCTTGTG<br>R: GACGACGAACAAAGGGCTTA	True
1500Gy-1	2	15,927,164	+1	hetero	T	F: ACTCACCGACGTGACTCTCA<br>R: TGGACACATGAAAAACCAAGA	True
1500Gy-1	1	12,333,406	Deletion	hetero	-22(-25+3)	F: TTCATTTCAGCTGCACATTCTT<br>R: AGGAGCAAGTGTGGAGAAGC	True
1500Gy-1	2	1,472,714	Deletion	hetero	-5	F: AAGTGCATTCAAAAATGGCTTT<br>R: CGAAGGCCTACAATATATGCAA	True
1500Gy-1	2	14,063,271	Deletion	hetero	-7	F: CTTCGGAACGCCCATAGTAG<br>R: TATGTCGGTTCGTGTTCTCG	True
1500Gy-1	3	18,575,840	Deletion	homo	-4	F: GAATCCGCAATACAATGTCG<br>R: CCAACATCGGATACAAACCA	True
1500Gy-1	1	17,443,382	Complex	hetero	SBSx2	F: TCCTGGATTCACTCCCAAAG<br>R: CTGGCAGTGACTGAACCAGA	True
1500Gy-1	1	25,439,405	Complex	homo	+2, SBS	F: GGAATCTTTTCACGCAGGTC<br>R: GGATTGATTCGGCAACCTTA	True
1500Gy-1	5<br><br>5	22,339,330<br><br>22,401,088	SV	hetero	~62 kbp inversion	F: AGCGAAGAACTCGACACTCC<br>R: GATCATGCGGTGATAAGCAA<br>F: TTGAATTTGCCAACTAACATTGA<br>R: GCCTACTCGGATTGGTGAAA	True
Details are shown in Supplemental Figure 6.							

* Originally identified as a 9-base insertion (a 25-base deletion accompanied by a 34-base insertion of unknown sequence), but the verification with Sanger sequencing showed that the actual length of insertion was 5 bp (a 25-base deletion accompanied by a 30-base insertion of unknown sequence).
